# Tris(pentafluorophenyl)borane-Catalyzed Reactions Using Silanes

**DOI:** 10.3390/molecules24030432

**Published:** 2019-01-25

**Authors:** Taylor Hackel, Nicholas A. McGrath

**Affiliations:** Department of Chemistry and Biochemistry, University of Wisconsin–La Crosse, La Crosse, WI 54601, USA; hackel.taylor@uwlax.edu

**Keywords:** tris(pentafluorophenyl)borane, silane, carbonyl reduction, Lewis acid, Si-H activation, mechanism, stereoselective

## Abstract

The utility of an electron-deficient, air stable, and commercially available Lewis acid tris(pentafluorophenyl)borane has recently been comprehensively explored. While being as reactive as its distant cousin boron trichloride, it has been shown to be much more stable and capable of catalyzing a variety of powerful transformations, even in the presence of water. The focus of this review will be to highlight those catalytic reactions that utilize a silane as a stoichiometric reductant in conjunction with tris(pentafluorophenyl) borane in the reduction of alcohols, carbonyls, or carbonyl-like derivatives.

## 1. Introduction

The utility of boron chemistry has exploded during the past 20 years, primarily due to the discovery and exploitation of tris(pentafluorophenyl)borane, B(C_6_F_5_)_3_. Commonly known as BCF, tris(pentafluorophenyl)borane [1,2,3,4,5,6,7] was found to have widespread use due to its unique reactivity, selectivity, and relative stability in a variety of reaction conditions. BCF has been used in: boration [8], hydrogenation [9,10], polymerization [11,12], hydrosilylation [13], alcohol or carbonyl deoxygenation [14], and Lewis acid catalysis [15], to name a few. BCF has also been shown to promote the selective chlorination of silanes using HCl to give both monochloro- and dichlorosilanes [16]. Before the discovery of BCF, deoxygenation reactions utilized other less reliable boron Lewis acids such as BF_3_ in conjunction with silanes [17,18,19,20,21,22]. BCF was also at the heart of the “frustrated Lewis pair” (FLP) [23] chemistry movement which bridged the gap between organic and inorganic chemistry in its utility. The strong Lewis acidic nature of BCF is based on the highly electron-withdrawing pentafluorophenyl groups attached to an already electron-deficient boron atom. BCF was first synthesized in 1963 [24,25], although it did not find widespread use until recently. A direct experimental comparison of BCF to other common B-based Lewis acids, BCl_3_ and BF_3_, concluded that BCF was comparable to BF_3_ in terms of its relative Lewis acidity [26]. Although similar in Lewis acid strength, BCF was found to be far more versatile in terms of its relative stability and functional group tolerance.

The term “frustrated Lewis pair” (FLP) has become associated with any molecule having high Lewis acidity combined with large steric bulk that renders it incapable of forming standard acid-base adducts [27]. This was a phenomenon first observed by H. C. Brown when he showed that lutidine formed an adduct with BF_3_ but not with the less Lewis acidic and bulkier B(Me)_3_ [28]. This unique combination enables FLPs to engage with reagents such as dihydrogen [4,5], alkenes [29,30], alkynes [31], carbon dioxide [32], and carbon monoxide [33,34,35]. This chemistry has found widespread utility throughout synthetic organic, organometallic and inorganic chemistry.

One of the many versatile uses of BCF has been in the total synthesis of natural products in which standard Lewis acids failed to produce the desired reaction outcomes. One example of this was in Njardarson’s formal synthesis of platensimycin [36]. The unique reactivity of BCF was used to promote a challenging deprotection of an elaborate aryl methyl ether. The free phenol was required to undergo a phenoxy-promoted alkylative dearomatization reaction to give the desired polycyclic framework of the natural product. A second example was an equally elaborate aryl methyl ether deprotection in a synthetic approach toward a family of [3.3.1]-bicyclic phloroglucinol-derived natural products, which failed under traditional Lewis acid conditions [37]. When BCF was employed with triethyl silane, the methyl aryl ether was selectively deprotected to give a free phenol. This phenol was needed for an oxidative dearomatization to give a cyclohexadieneone intermediate primed for a tandem bis-radical cyclization to give the [3.3.1]-bicyclic framework of the natural product family. BCF was also applied to the modification of complex bioactive compounds such as natural products and drugs to probe structure-activity relationships [38].

Another use of BCF has been to react various *O*-heterocycles in the presence of silanes to generate ring-opened allylic and homoallylic alcohols [39]. It was found that the allylic position of the *O*-heterocyclic compound was reduced preferentially without any measurable extent of olefin stereochemistry being lost. Substrates that were deemed electronically symmetric were found to rely on the steric hindrance of the groups that were found to dictate the regiochemistry of the reduction. An additional metal-free method for the BCF-promoted reactivity of heterocycles was reported by Shi and coworkers [40]. They found that BCF was catalytically competent to promote the reduction of various *N*-heterocycles.

A unique application for BCF has been the deoxygenation and reductive carbocyclization of saturated and unsaturated carbohydrates. Using a vast excess of silane, Gagne and coworkers were able to completely deoxygenate sugars to their corresponding hydrocarbon frameworks [41]. In a more controlled method the reaction could be tuned to produce both siloxy-cyclopropane and siloxy-cyclopentane derivatives [42]. The method also applied to a variety of biologically sourced polyols that could be converted into various chiral synthons [43].

## 2. Triethylsilane

### 2.1. Aryl and Alkyl Ethers

**Figure 1:**
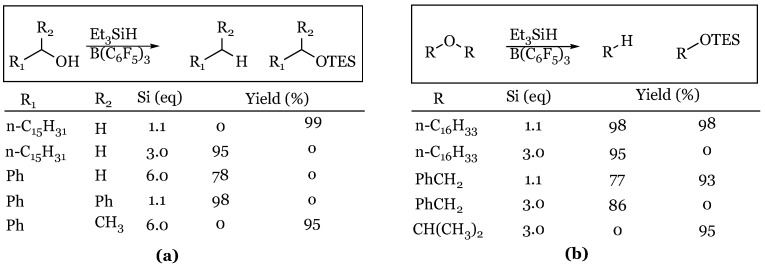
Representative reductions with triethylsilane: (**a**) alcohols; (**b**) ethers.

The first reported catalytic use of a Lewis acid for the reduction of alcohols and ethers with a silane occurred in 2000 by the groups of Gevorgyan and Yamamoto [44]. In the study, they reported the extent of reduction to be dependent upon the equivalents of triethylsilane used (Figure 1).

Primary alcohols produced TES ethers with 1.1 equivalents of silane and alkanes when an excess was used. A diaryl secondary alcohol was reduced directly to the alkane, while an aryl methyl secondary alcohol stopped at the silyl ether, regardless of the silane equivalents in each case. Symmetric ethers were cleaved to the alkane and silyl ether with 1.1 equivalents of silane and were fully reduced to alkanes with 3.0 equivalents. More sterically hindered ethers such as diisopropyl ether afforded exclusively the silyl ether, even with 3.0 equivalents of silane.

**Figure 2:**
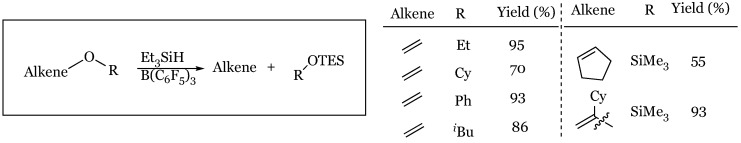
Representative reductions of enol ethers with triethylsilane.

The research group of Dobrovetsky was recently able to extend the reduction of ethers to include enol ethers [45]. Both alkyl enol ethers and silyl enol ethers were substrates for the reaction (Figure 2). The reaction produces the free olefin and either an alkyl silyl ether or bis-silyl ether.

### 2.2. Aldehyde, Acid Chloride, Ester, and Carboxylic Functions

**Figure 3:**
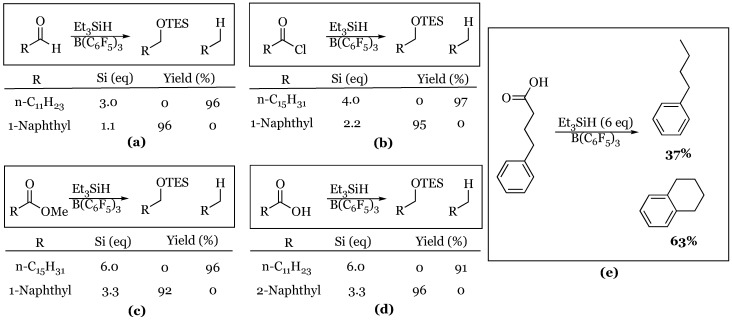
Representative reductions with triethylsilane: (**a**) aldehydes; (**b**) acid chlorides; (**c**) esters; (**d**) carboxylic acids; (**e**) 4-phenylbutyric acid reduction.

The Gevorgyan and Yamamoto groups extended their study with triethylsilane to include aldehydes, acid chlorides, esters and carboxylic acids the following year [46]. In all cases, it was shown that the aliphatic substrates were exhaustively reduced to hydrocarbons while the 1-naphthyl variants produced exclusively the silyl ether (Figure 3). A variety of different aliphatic carboxylic acids with phenyl groups appended were screened, and each produced the corresponding n-alkyl benzene with the exception of 4-phenylbutyric acid. In this case, an intramolecular Friedel-Crafts alkylation was observed, resulting in tetralin as the major product and the expected butyl benzene as the minor product.

**Figure 4:**
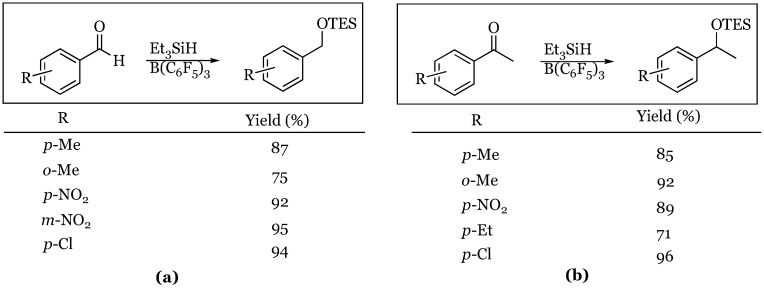
Hydrosilylation of aldehydes and ketones: (**a**) benzaldehyde derivatives; (**b**) acetophenone derivatives.

The substrate scope of aryl aldehydes was greatly expanded to include a variety of electron-rich and electron-deficient benzaldehyde derivatives (Figure 4) [47]. In addition, Laali and coworkers were able to demonstrate that a number of substituted acetophenone derivatives were substrates for hydrosilylation as well. All reactions were conducted with one molar equivalent of triethylsilane and therefore resulted in the isolation of the silyl ether product. The study went on to explore and compare the analogous reactions with various metal triflates as catalysts. The metal triflates caused a dramatic change in the chemoselectivity of the reaction. In these cases, they noted a substantial formation of the dibenzylether and benzylation of the solvent as a result of over-reaction.

### 2.3. Olefins

**Figure 5:**
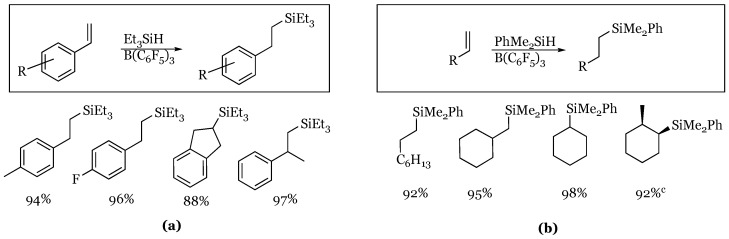
Hydrosilylation of olefins: (**a**) styrene derivatives with Et_3_SiH; (**b**) aliphatic systems with PhMe_2_SiH. ^c^ Product ratio cis: trans = 96:4.

The next advancement in the field involved the borane-catalyzed, *trans*-selective hydrosilylation of olefins [13]. The Gevorgyan group found that a variety of silane reagents were tolerated and the regiochemistry of the reaction suggested a direct addition of a silylium species across the olefin followed by subsequent trapping with a borohydride (Figure 5). The study involved olefins with a variety of substitution patterns modeling different electronic and steric preferences. The styrene derivatives proceeded in very high yields using triethylsilane while the aliphatic olefins required the use of dimethylphenylsilane to achieve comparable outcomes. The reaction of 1-methylcyclohexene afforded almost exclusively cis product *via* anti addition of the silyl and hydride components. The only aliphatic olefin shown to react with triethylsilane was the TIPS ether of 3-buten-1-ol, which resulted in an 87% yield of the expected hydrosilylated product. 

### 2.4. Carboxylic Acids

**Figure 6:**
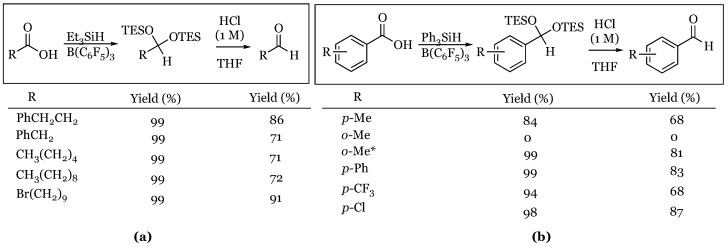
Conversion of carboxylic acids into aldehydes: (**a**) aliphatic carboxylic acids; (**b**) aromatic carboxylic acids; * Triethylsilane used in place of triphenylsilane.

Recently Brookhart and coworkers greatly extended the substrate scope of the reaction to include carboxylic acids [48]. They were able to demonstrate that both aliphatic and aromatic carboxylic acids could be efficiently converted into their corresponding disilyl acetals (Figure 6). They also found optimal results were found by using tertiary silanes and that mild acidic workup cleanly afforded the aldehyde product. It was found that making the change to triphenylsilane was required to attain reasonable yields in the case of aryl carboxylic acids. The exception for this was *o*-toluic acid, in which triphenylsilane did not produce any measureable product while triethylsilane worked well.

### 2.5. Sulfides and Dithianes

**Figure 7:**
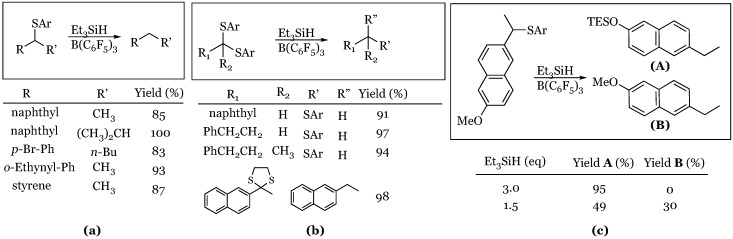
Hydrodesulfurization of carbon-sulfur bonds: (**a**) sulfides; (**b**) dithianes; (**c**) sulfide versus methyl aryl ether competition reactions.

An extension of the utility of these reaction conditions was realized in 2015 by Akiyama and coworkers when they demonstrated the hydrodesulfurization of sulfides and dithianes [49]. In a reaction that could be considered analogous to a Raney-Ni reduction, they were able to accomplish desulfurization of a variety of sulfides to yield the corresponding hydrocarbons. They were also able to reduce a number of dithianes to produce either the sulfide or fully desulfurized hydrocarbon, depending on substrate (Figure 7). By contrast, when the thioacetal was derived from naphthaldehyde rather than acetonaphthone, the sulfide was isolated rather than hydrocarbon.

### 2.6. Amides and Nitriles

**Figure 8:**
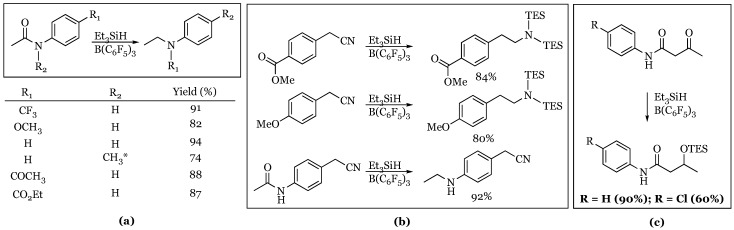
Selectivity experiments with various functional groups: (**a**) Reduction of acetanilide amides in the presence of ethers, ketone, and esters; (**b**) reduction of benzyl nitriles in the presence of ethers, esters; (**c**) acetoacetanilide reductions. * diethylsilane was used.

The utility of the borane-silane reduction was also shown to be applicable to acetanilide amides and benzylic nitriles by McGrath and coworkers [50]. Acetanilide amides were conveniently reduced to their corresponding secondary aniline derivatives (Figure 8). Tertiary aniline derivatives were possible, but required the use of the more reactive diethylsilane to afford reasonable yields. The selectivity of the reduction was also examined, and it was determined that acetanilide amides were reduced preferentially over acetophenone or ethyl benzoate derivatives. Benzylic nitriles were also reduced to the bis-silyl-protected amines. The nitriles were found to be more reactive than methyl aryl ethers and esters, but the acetanilide amide was reduced in preference to the nitrile. In a mechanistic approach, acetoacetanilide derivatives were screened and shown to result in ketone reduction to the silyl ether with no reduction of the amide even with a large excess of silane and catalyst.

### 2.7. Sulfoxides and Sulfones to Sulfides

**Figure 9:**
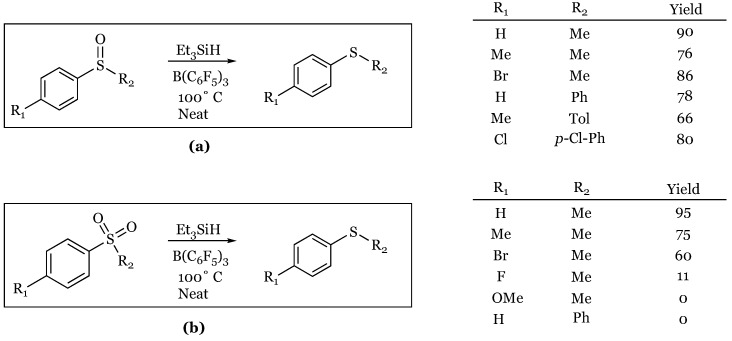
Reduction of (**a**) sulfoxides and (**b**) sulfones to sulfides.

The reduction of both sulfoxides and sulfones to their corresponding sulfides in the absence of solvent was discovered last year by Oestreich and coworkers [51]. The reactions are run neat at 100 °C for 8 hours and require using excess triethylsilane to achieve high yields (Figure 9). The reactions were shown to work in toluene, but in far inferior yields.

## 3. Triphenylsilane

### 3.1. Aromatic Aldehydes, Ketones, and Esters

**Figure 10:**
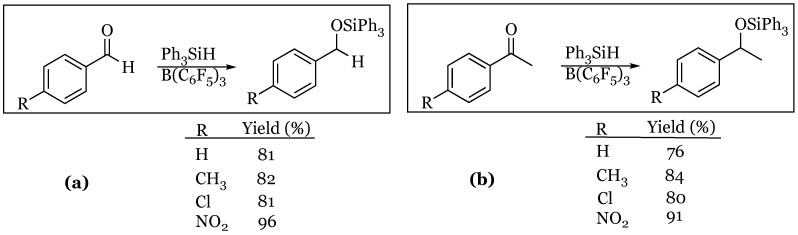
Reduction of: (**a**) benzaldehyde derivatives; (**b**) acetophenone derivatives.

The catalytic reduction of benzaldehyde and acetophenone derivatives with triphenylsilane was also explored in 1996 by Piers [52]. In this study, one equivalent of silane was used with 2 mol % catalyst to afford the silyl ether selectively, regardless of the para substituent (Figure 10). This was one of the initial studies into the unique mechanism of the reaction, which will be discussed further in this review. Both the aryl aldehydes and ketones were shown to be selectively reduced to the silyl ether in high yield, with the highest yielding reactions being the *p*-nitro derivatives in both cases. The authors went on to show that under similar conditions, ethyl benzoate was reduced to benzaldehyde, presumably by subsequent hydrolysis of the initial mixed silyl-ethyl acetal produced.

### 3.2. Silylation of Alcohols

**Figure 11:**
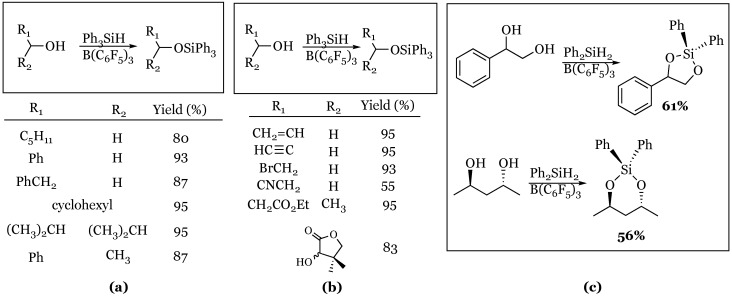
Alcohol and diol reactivity: (**a**) Silylation of primary and secondary alcohols; (**b**) silylation of alcohols in the presence of reactive functional groups; (**c**) silylation of 1,2-, and 1,3-diols with diphenyl silane.

Primary and secondary alcohols were shown by Piers to be substrates for catalytic silylation using triphenylsilane to produce triphenylsilyl ethers [53]. The reaction was shown to be tolerant of alkenes, alkynes, halogens, nitriles, esters, and lactones (Figure 11). The study then investigated 1,2-diols and 1,3-diols with diphenylsilane and demonstrated both were substrates for the reaction resulting in five- and six-membered ring silyl acetals respectively, in reasonable yields. They also demonstrated that a variety of substituted phenols were substrates for the reaction as well.

## 4. Diphenyl Silane

### 4.1. Phosphonic and Phosphinic Esters

**Figure 12:**
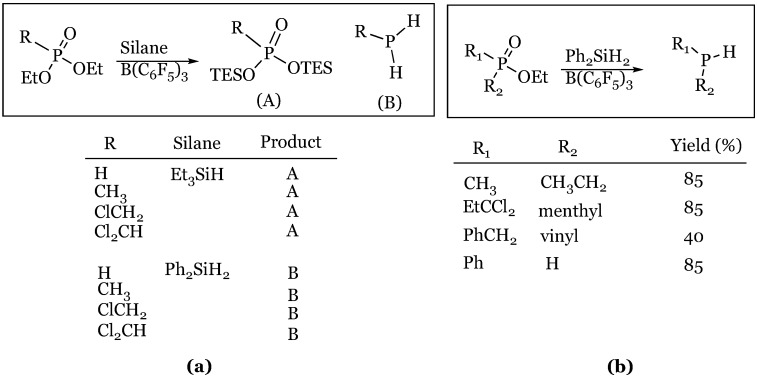
Reduction of phosphorous compounds: (**a**) Phosphonic esters; (**b**) Phosphinic esters.

A completely unique mode of reaction was explored by Keglevich and coworkers when they showed the silane/borane system was capable of reducing phosphorus-containing compounds with varying oxidation states of phosphorus [54]. The reaction of phosphonic and phosphinic esters with a hydrosilane and tris(pentafluorophenyl)borane was shown to produce either the bis-silylated phosphonate or free phosphine, depending on silane used (Figure 12). The comparable reaction of phosphinates (Figure 12b) proceeded with only 1 equivalent of phenylsilane and resulted in the corresponding secondary phosphine in reasonable yield. It appeared that the vinyl phosphinate caused issues resulting in a drastically diminished yield of the desired product.

### 4.2. Indoles, Enamines, Cinnamic Acid, Isocyanates, and Enol Ethers

**Figure 13:**
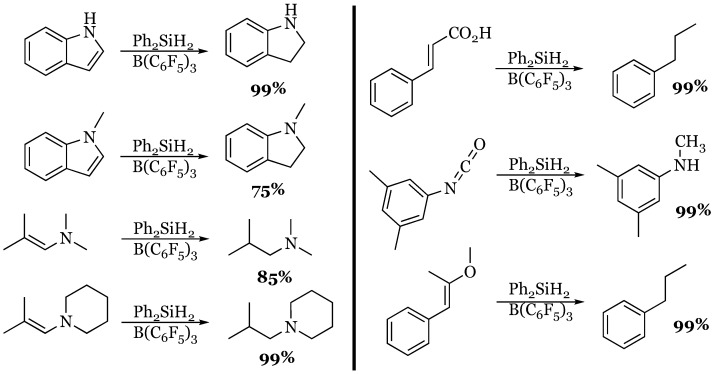
Reduction of indoles, enamines, cinnamic acids, isocyanates, and enol ethers.

The substrate scope of reduction was greatly expanded using diphenyl silane by Zhang to include indoles, enamines, cinnamic acid, isocyanates, and enol ethers [55]. The indoles and secondary enamines were efficiently reduced to the corresponding indolines and tertiary amines in high yield (Figure 13). Cinnamic acid was fully reduced to the hydrocarbon propyl benzene, while an isocyanate was reduced to a methyl amine, and an enol ether of phenylacetone was deoxygenated to give propyl benzene. 

### 4.3. Disproportionation Reaction of Indoles

**Figure 14:**
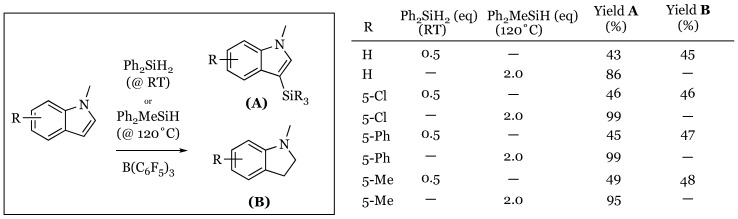
Convergent disproportionation of indoles.

Last year, Zhang and coworkers further explored the reactivity of indoles with tris(pentafluorophenyl)borane and diphenylsilane [56]. A variety of substituted indoles were subjected to the reaction conditions using diphenylsilane. Depending on whether 0.5 or 2.0 molar equivalents of silane were used, the products were found to correspond to roughly equal amounts of the indolines and silylated indoles or to solely silylated indoles, respectively (Figure 14). Regardless of substituent or substitution pattern (not shown but included in original work), the yields were nearly quantitative in both cases.

### 4.4. Regioselective Deoxygenation of 1,2-Diols

**Figure 15:**
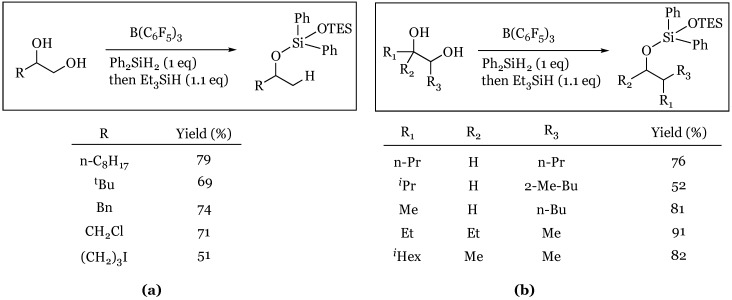
Reaction of: (**a**) Terminal 1,2-Diols; (**b**) Internal 1,2-Diols.

The Morandi group demonstrated that terminal 1,2-diols react regioselectively reducing the primary alcohol to yield the secondary silyl ether [57]. A variety of terminal 1,2-diols were subjected to BCF and diphenyl silane followed by addition of triethylsilane, and each resulted in reduction of the primary alcohol and protection of the secondary alcohol (Figure 15). The same group went on to explore internal 1,2-diols and found that under analogous reaction conditions, a reductive pinacol-type rearrangement was observed [58]. They also noted that the reaction proceeds through a concerted, stereoinvertive mechanism giving rise to highly enantiomerically enriched products.

## 5. Phenylsilane

### 5.1. Cyclic Imides

**Figure 16:**
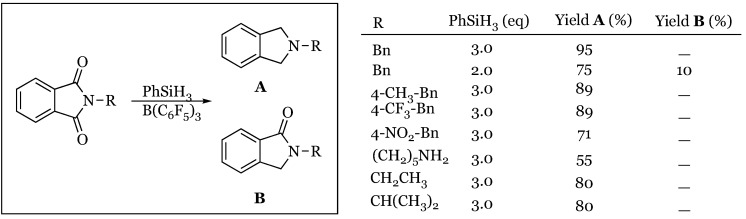
Mild and selective formation of *N*-substituted pyrrolidines from cyclic imides.

The synthesis of *N*-substituted pyrrolidines was realized this year by Xie and coworkers directly from the corresponding cyclic imide using phenylsilane as the stoichiometric reductant [59]. A variety of *N*-substituted phthalimide derivatives were subjected to 2-3 equivalents of phenylsilane and catalytic borane to produce the *N*-substituted pyrrolidines (Figure 16). When the equivalents of silane were reduced, a small amount of the lactam remained without any measurable hemiaminal-type products present. Therefore, it can be concluded that in the two-step reduction of each carbonyl group of the cyclic imide, the first step is rate determining. To date, this represents one of the most mild and selective methods of producing substituted pyrrolidines.

### 5.2. Deoxygenation of Sulfoxides and Amine N-Oxides

**Figure 17:**
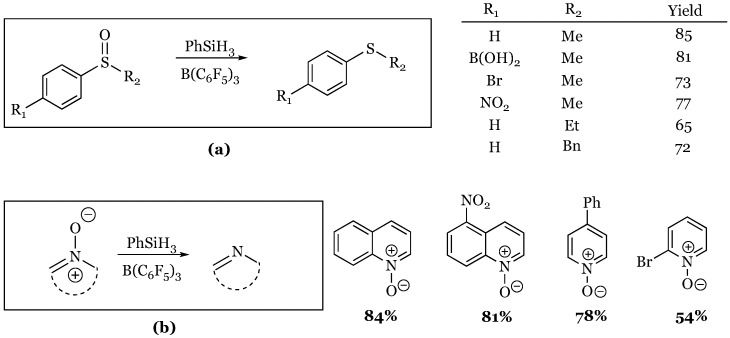
Deoxygenation of sulfoxides and amine *N*-oxides: (**a**) Aryl sulfoxides; (**b**) Aryl-N-oxides.

Aryl methyl sulfoxides and heterocyclic aromatic *N*-oxides were shown to be substrates for deoxygenation using the highly reactive phenylsilane and BCF [60]. The aryl component of the sulfoxide tolerated a wide variety of functionalization and the *N*-oxides tolerated nitro groups and halogens (Figure 17). A few non-aromatic substrates were also shown to work in both cases, but with diminished yields.

## 6. Diphenylmethylsilane

### Enones and Silyl Enol Ethers

**Figure 18:**
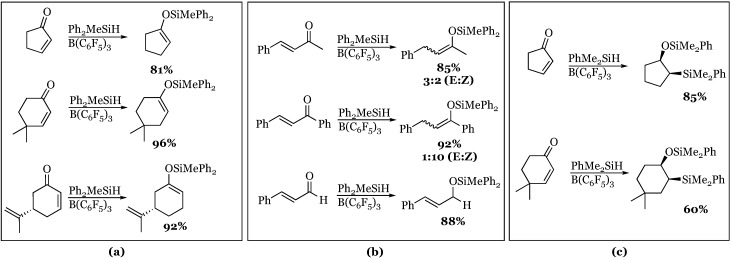
α,β-Unsaturated ketone reactivity: (**a**) Reduction of cyclic enones; (**b**) reduction of acyclic enones and cinnamaldehyde; (**c**) iterative stereoselective silylation of enones.

The reduction of α,β-unsaturated aldehydes and ketones by diphenylmethylsilane was explored [61]. All cyclic enones proceeded smoothly to their corresponding cyclic silyl enol ethers, without any measurable side products formed, even in the presence of an isolated alkene (carvone) (Figure 18). The acyclic enones produced the silyl enol ether and in both cases favored the *Z*-alkene product. In the case of cinnamaldehyde, the styrene silyl ether product was isolated rather than the enol ether. Other substrates with increased steric bulk at the β-position also gave measurable amounts of 1,2-hydrosilylation product much like cinnamaldehyde. Using the more reactive dimethylphenylsilane (discussed later), enones were shown to undergo iterative hydrosilylation reactions to give *cis*-β-siloxyalkylsilanes in high yields, although they tended to require long (40 h) reaction times.

## 7. Dimethylphenylsilane

### 7.1. Reductive Amination

**Figure 19:**
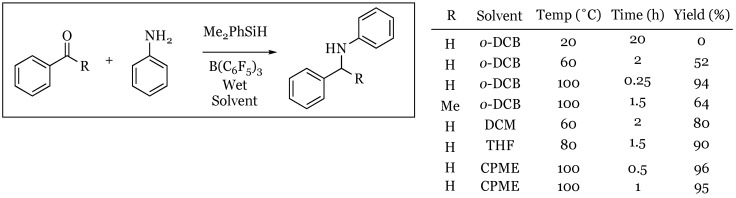
Reductive amination of aniline and benzaldehyde (acetophenone) with various solvents, temperatures, and reaction times to produce *N*-benzyl aniline products.

The borane/silane system was also found to be capable of promoting the reductive amination of aniline and benzaldehyde with a variety of wet solvents (Figure 19). The process of reductive amination requires a catalyst that is tolerant of water due to its production as a byproduct of the process. Most Lewis acid boron catalysts therefore fail due to their incompatibility with water. The water tolerance of BCF was first discovered by Yamamoto’s group [62] and recently Ingleson and coworkers [63] were able to show that BCF is uniquely capable of promoting a reductive amination. They extended the reaction to a ketone, acetophenone, and still reported a reasonable yield of product. This represents a mild alternative to sodium cyanoborohydride-promoted reductive amination reactions. 

### 7.2. Conjugated Esters and Amides Leaving Carbonyl Groups Intact

**Figure 20:**
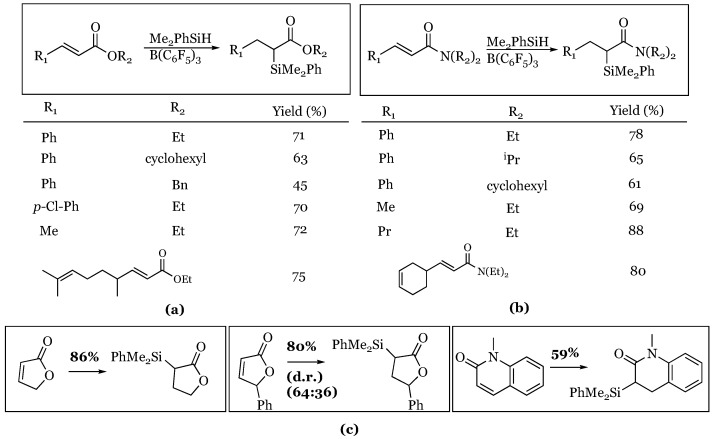
Selective conjugate reduction of α,β-unsaturated esters (**a**); amides (**b**); lactones/lactams (**c**).

Unsaturated esters and amides, both acyclic and cyclic were shown to be substrates for reduction, and in all cases, the carbonyl functionality remained intact, while the alkene was reduced [64]. One substrate from each class was screened that had an isolated alkene to demonstrate the regiochemistry of the reaction (Figure 20). Three butenolide lactones and a 5,6-dihydropyridone-lactam were shown to follow the expected reactivity pattern to generate the α-silyl lactone and lactam respectively. 

### 7.3. Ring Opening and Closing Cascades of Furans

**Figure 21:**
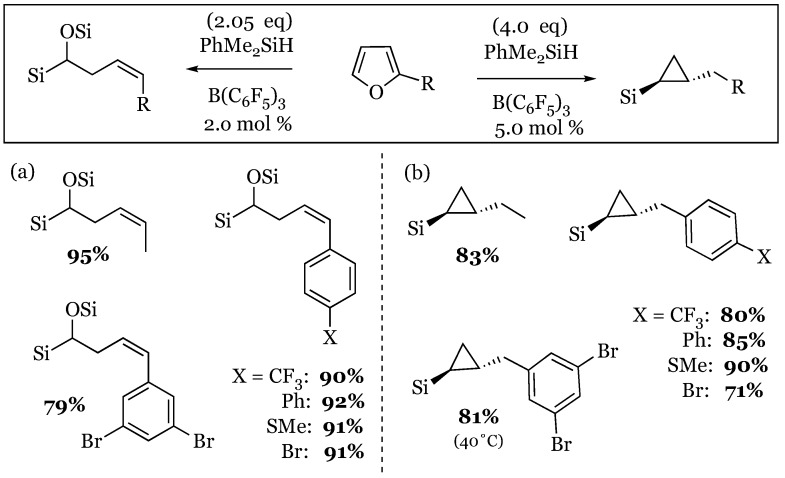
Conversion of furans to: (**a**) α-siloxy-(*Z*)-alkenylsilanes; (**b**) alkylcyclopropylsilanes.

A variety of substituted furans were shown by Chang and coworkers to be substrates for a two-step, ring opening and ring closing process [65]. The reaction could be controlled based on catalyst loading and silane equivalents used (Figure 21). This method provides a reliable method to access α-siloxy-(*Z*)-alkenylsilanes and anti-(2-alkyl) cyclopropylsilanes, both of which are useful synthetic intermediates that can be further elaborated.

## 8. Diethylsilane

### 8.1. Nitriles to Generate Primary Amine Salts

**Figure 22:**
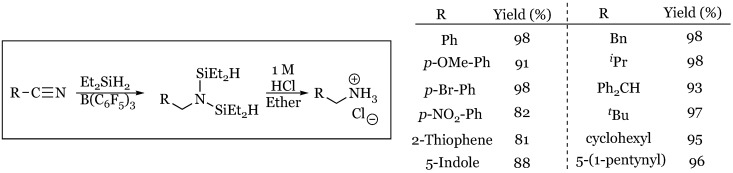
Two-step reduction and deprotection of aryl and alkyl nitriles to their corresponding primary amine hydrochloride salts using diethylsilane and 1 M HCl.

A mild extension of the method to reduce nitriles to their corresponding primary amines was discovered in 2015 by Chang and coworkers [66]. A variety of aryl and alkyl nitriles were screened and all proceeded smoothly to the bis-silyl protected amine that was hydrolyzed with dilute aqueous HCl to produce the hydrochloride salt of the primary amine (Figure 22). All nitriles screened proceeded in greater than 80% yield and displayed tolerance for functionalities such as NO_2_, halogens, heterocyclic aromatics, aryl silyl ethers, cyclic alkenes, and alkynes. In select cases the silyl imine could be isolated when 1.0 equivalents of silane were used. 

### 8.2. Internal Ynamides Leading to β-Silyl (Z)-Enamides 

**Figure 23:**
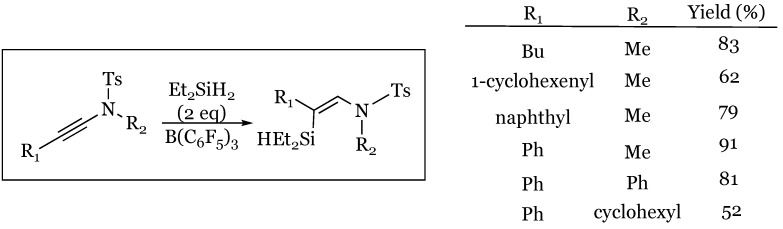
Hydrosilylation of internal ynamides to give silyl enamides.

The substrate scope was further advanced when Chang and coworkers discovered that internal ynamides, the close relative to the enamine, were also susceptible to reduction using this catalytic system [67]. In their study, they examined a variety of sulfonamide protected ynamides and noted a unique but reproducible regiochemistry for the reduction (Figure 23). In all cases, regardless of alkyne or amine substituent, the reduction occurred such that the silyl group was added onto the β-carbon rather than the typical α-carbon for enamines. Additionally, they noted the exclusive formation of the (*Z*)-enamide product, attributed to the β-silicon effect on their postulated ketene iminium intermediate in the process. 

## 9. *n*-Butylsilane

### Polycarboxylic Acids into Their Corresponding Alkanes

**Figure 24:**
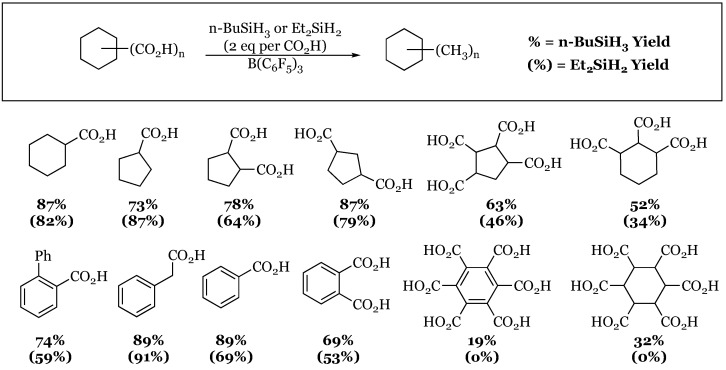
Reduction of mono-, di-, tri-, tetra-, and hexacarboxylic acids to methyl groups. * Use of *n*-butylsilane resulted in a 19% and 32% yield of the desired product, respectively.

The ability of this catalytic system to reduce carboxylic acids was truly tested by the McRae group when they decided to investigate the reduction of substrates bearing more than one carboxylic acid functionality (Figure 24) [68]. The study explored a wide variety of poly-carboxylic acid substrates in which the only notable substrates that resisted reduction were mellitic acid (1,2,3,4,5,6-benzenehexacarboxylic acid) and the non-aromatic variant 1,2,3,4,5,6-cyclohexanehexacarboxylic acid. Yields varied widely depending on substrate, but most reactions achieved yields of around 70% or better. Yields of the transformations were all GC based, and therefore could be considered to be higher than the yield that might be obtained following isolation.

## 10. Tetramethyldisiloxane

### Secondary and Tertiary N-Phenyl Amides

**Figure 25:**
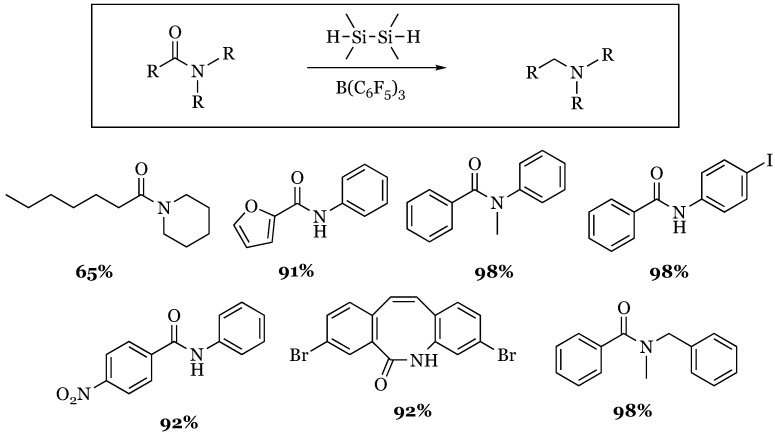
Tetramethyldisilane reduction of select amides with tris(pentafluorophenyl)borane.

A wide variety of secondary and tertiary amides were successfully reduced to their corresponding amines with tetramethyldisilane by Adronov and coworkers in 2014 [69]. A variety of substituted aromatic, heteroaromatic, cyclooctyne, and alkyl amides were examined and were found to generally good substrates for reduction. (Figure 25). Only one amide in which both the carbonyl and amino groups were alkyl-based that gave a reasonable yield was included and resulted in a 65% yield. A similar study by Cantat confirmed that aliphatic amides were not reliable substrates for reduction [70]. There were six additional substrates included in the study that did not produce any of the desired product, indicating that amides remain to be challenging substrates for this reduction.

## 11. Polymethylhydrosiloxane

### Aldehydes and Ketones

**Figure 26:**
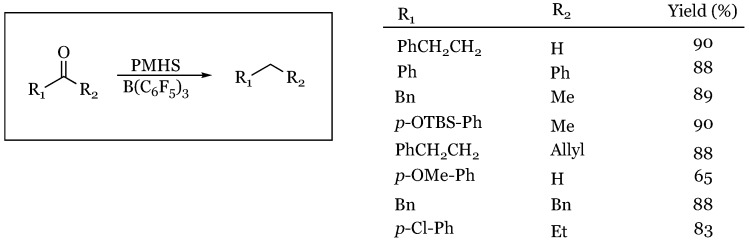
Defunctionalization of carbonyl groups using polymethylhydrosiloxane.

The Chandrasekhar group made use of a polymeric silane (polymethylhydrosiloxane) (PMHS) to promote the defunctionalization of various carbonyl groups to their corresponding methylene groups [71]. They found that both aromatic and aliphatic aldehydes and ketones were reduced to their corresponding alkanes in very high yields (Figure 26). In general, the reaction was tolerant of most functional groups. Benzylamide was unreactive under any of the conditions screened indicating that amides, even sterically accessible amides, are challenging under these conditions.

## 12. Stereoselective Reactions

### 12.1. σ-π Chelation-Controlled Stereoselective Hydrosilylation of Ketones

**Figure 27:**
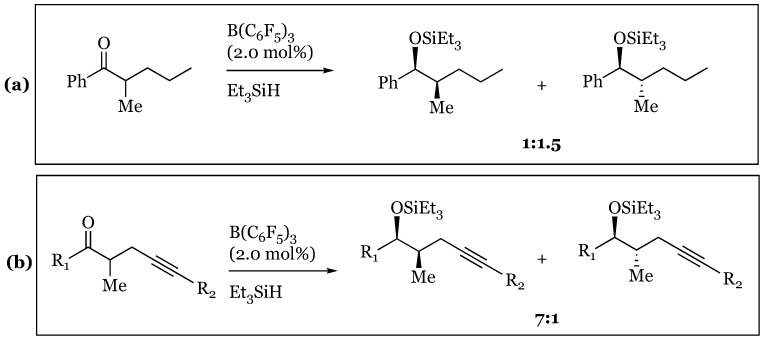
Diastereoselectivity: (**a**) without sigma-pi chelation; (**b**) with sigma-pi chelation. R_1_ = Ph and R_2_ = H.

**Figure 28:**
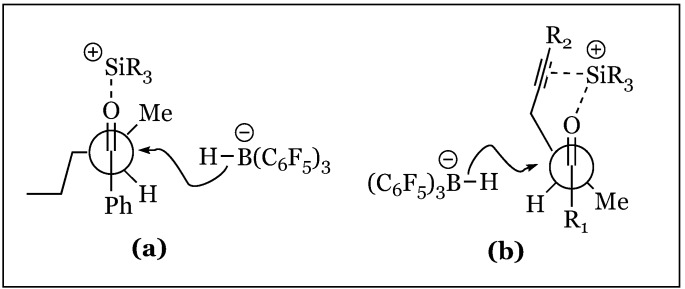
Felkin-Ahn models for: (**a**) propyl; (**b**) propargyl substituted reactants.

1,2 Asymmetric induction has been used in the observation of a slight preference for the *anti*-conformation (*syn/anti* 1:1.5) in the hydrosilylation of 2-methyl-1-phenylpentan-1-one (Figure 27) [72,73]. The Felkin-Ahn model justifies predominant hydride transfer to the Si face of the prochiral ketone in which the propyl group is assigned as the largest substituent (Figure 28a). The low selectivity is attributed to the small relative size difference between the propyl and methyl α-substituents. Surprisingly, treatment of 2-methyl-1-phenylpent-4-yn-1-one under the same conditions (2 mol% B(C_6_F_5_)_3_ and Et_3_SiH), resulted in predominant formation of the *syn* isomer (*syn/anti* 7:1). The unanticipated reversal in diastereoselectivity might originate within σ−π chelation of the β alkynyl and carbonyl lone pairs to a bridging R_3_Si cation. A six-membered transient intermediate is hypothesized to undergo hydride attack on the less hindered face, resulting in the syn isomer (Figure 28b).

**Figure 29:**
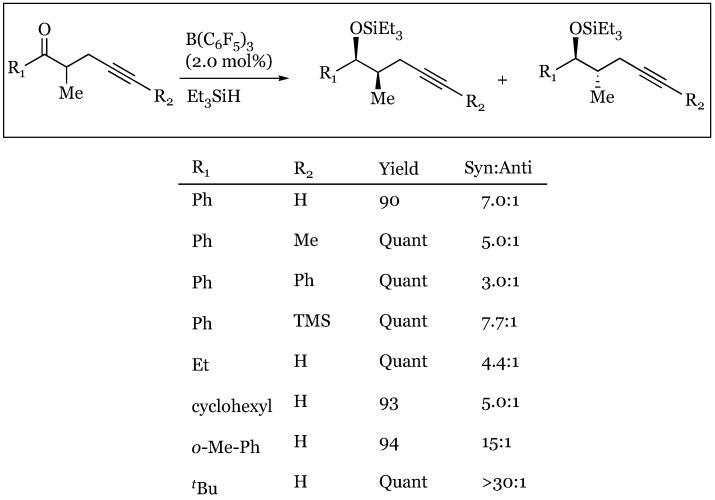
*Syn* selectivity and yield based on ketone and alkyne substitution.

**Figure 30:**
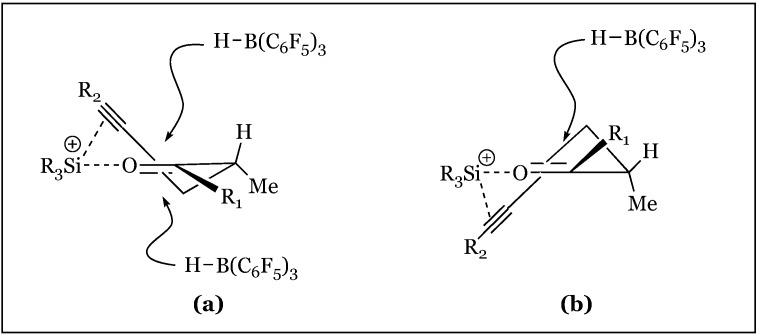
Half-chair conformations explaining *syn*-selectivity: (**a**) relatively small R_1_ groups do not distort the carbonyl conformer framework; (**b**) increasing the size of R_1_ causes a conformational change of Me group favoring attack from the top, increasing *syn* selectivity.

Stereoselectivities were decreased when bulkier groups were appended to the propargylic site, apparently diminishing the chelating interactions (Figure 29). In contrast, increasing the size of the substituent appended to the keto carbon enhanced *syn* selectivity (15:1 for o-MePh and >30:1 for ^t^Bu). This effect is attributed to increased steric repulsion with the α-methyl, shifting its position *anti* to R_1_ and occluding hydride attack from this face (Figure 30). Asymmetric induction was also induced in the 1,3 system of alkynyl ketones, with preferential formation of the *anti*-product.

### 12.2. α-Diketones to Silyl-Protected 1,2-Diols

**Figure 31:**
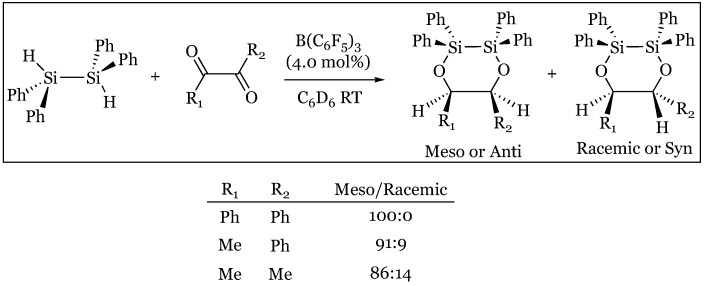
*Meso* selectivity of α-diketones reacted with tetraphenyldisilane.

**Figure 32:**
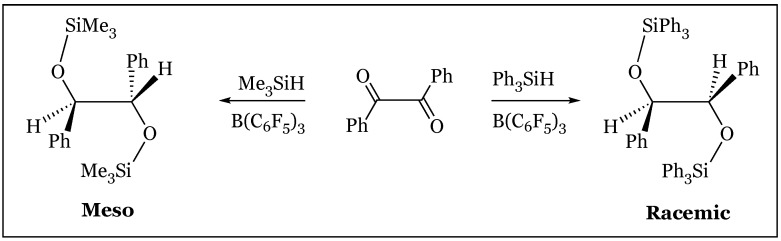
Stereoselectivity of α-diketone silylation product based on silane.

Stereoselectivity may conversely be manipulated in the selection of silane rather than substrate characteristics, as the bis(hydrosilylation) of α-diketones forms the *meso* (*anti*) or *dl* (*syn*) product based on steric constraints about the silane center [74,75]. Treatment of α-diketones with the bridged tetraphenyldisilane formed nearly exclusively the *meso* product, with the highest selectivity observed within the most sterically hindered bisphenyl ketone (Figure 31). Steric hindrance encountered in ring formation of the bis-oxygen bridged product arguably favors the observed stereoselectivity. The Cram-chelate model may also be relevant, as hydride attack from the hydridoborate favors the H side of a six-membered intermediate including the bridging Ph_2_Si-SiPh_2_ chelator. Monosilanes are also subject to analogous steric concerns, as Me_3_SiH preferred the meso product while Ph_3_SiH preferred the racemic stereoisomer (Figure 32).

**Figure 33:**
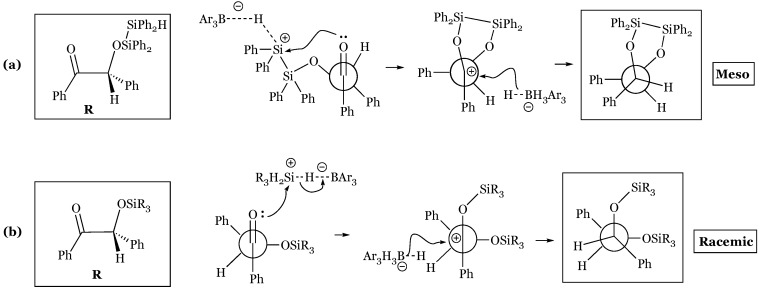
Felkin-Ahn models for second silyl addition with an α-diketone using: (**a**) disilane; (**b**) “large” monosilane placed orthogonal.

These results were based on the relative size of the first silyl ether addition, as larger silanes were ranked as the largest substituent and placed orthogonal in the Felkin-Ahn model, while smaller silanes were superseded by substituents of the substrate (Figure 33b).

## 13. Mechanistic Studies

### 13.1. Silane Activation Mechanism

**Figure 34:**
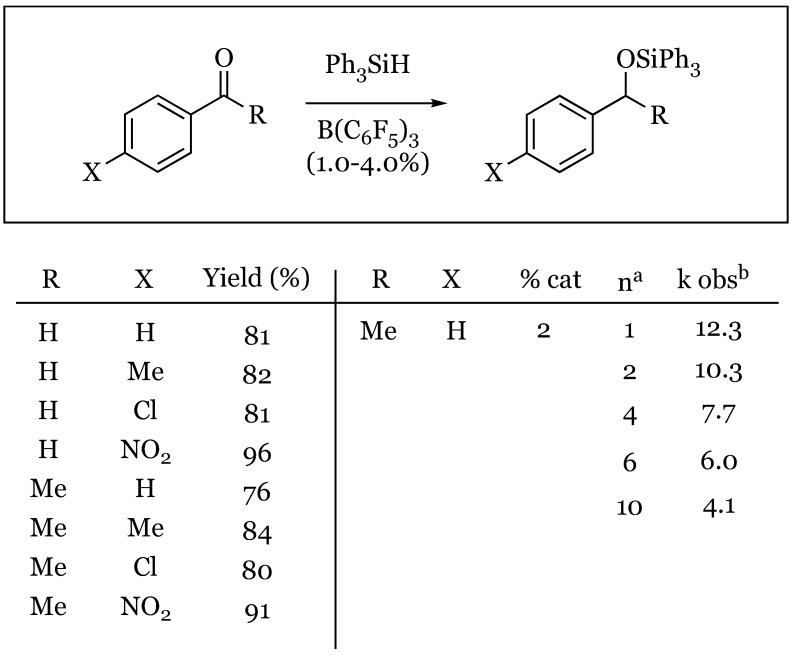
Hammett study of aromatic aldehydes and ketones. ^a^ For *n* = 1, concentration = 0.385 M; ^b^ First-order rate constants.

Early mechanistic exploration of the B(C_6_F_5_)_3_-silane catalytic reduction of carbonyl functionalities by Piers and Parks yielded unprecedented results [52]. Nucleophilic activation of the Si-H bond concurrent with electrophilic catalytic activation of the carbonyl oxygen had been observed within representative systems of amide or halide nucleophiles paired with a Lewis acid such as ZnCl_2_ or BF_3_·Et_2_O. However, the observed reactivity series ethyl benzoate >> acetophenone > benzaldehyde, in which reactivity decreased parallel to base strength, countered the assumed catalytically pertinent dative interaction between the carbonyl’s lone pair and the tris(pentafluoro-phenyl)borane catalyst. These results were further reinforced by an increase in rate proportional to the degree of electron withdrawing capabilities of X-substituted aromatic carbonyls in a Hammett study, as well as by a decrease in the rate constant upon increase in carbonyl substrate concentration (Figure 34). Considering the reaction was determined to be first order in silane, Piers, et al. inferred a novel role for the tris(pentafluorophenyl)borane catalyst. Their hypothesis required the “liberation” from the basic carbonyl group in which the Lewis acid catalyst activates the Si-H bond for nucleophilic attack from the carbonyl substrate. 

**Figure 35:**
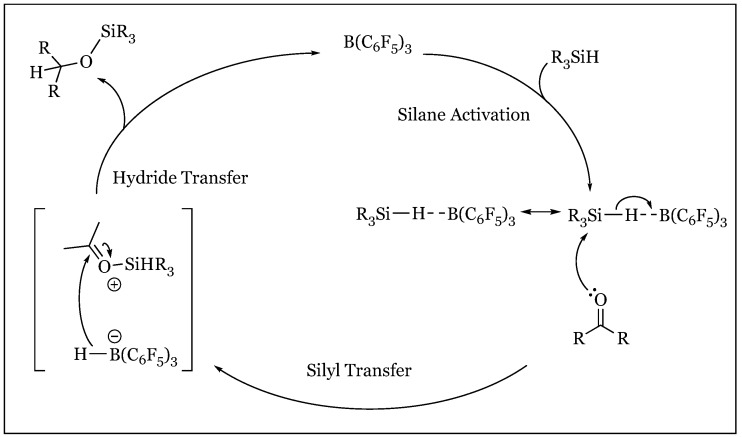
Piers mechanism of tris(pentafluorophenyl)borane catalyzed hydrosilylation of carbonyl functionalities.

Continuation of previous mechanistic inquiry culminated in a new proposed mechanism (Figure 35) initiated with Si-H coordination to the boron center of the Lewis Acid catalyst [76]. The complex is subsequently attacked by the substrate as hydride is transferred from silane to boron, garnering an “incipient” silyloxonium ion paired with a hydridoborate counter anion. Reduction is fulfilled in hydride transfer from the hydridoborate to the carbonyl carbon, electrophilically enhanced due to silyl activation of the carbonyl oxygen. Evidence for a silane-borane complex was procured in the observed complete protium/deuterium exchange upon mixture of 1:1 Et_3_SiH and Ph_3_SiD with 10% B(C_6_F_5_)_3._ Additionally, a primary kinetic isotope effect of 1.40 noted upon treatment of acetophenone with catalyst and Ph_3_SiX (X = H/D) agrees with the proposed hydride withdrawal as Si-H hydride abstractions have produced isotope effects of 1.4–1.9. Nucleophilic attack of the borane/silane complex was deduced based on competition reactions in which the more basic substrate was preferentially reduced at significant ratios of >96:1; for example, benzaldehyde or acetophenone with less basic ethyl benzoate. However, it must be noted that the more basic substrate proceeds at a slower rate independently, presumably due to the deleterious effects of borane adduct formation. While the source of hydride reduction may have originated from either unreacted silane or the hydridoborate ion, the latter was confirmed. Under controlled conditions in which silane was presented as a sole source of hydride, an inconsistent product distribution was generated, suggesting the hydridoborate as the ultimate reductant. In these alternative reductive systems, catalytic activity was engendered by silylium in the presence of a borate counterion, effectively disabling its potential to act as a Lewis acid hydride recipient: (Et_3_Si)^+^(B(C_6_F_5_)_4_)^−^, (Ph_3_C)^+^(B(C_6_F_5_)_4_)^−^, or 1,2-(B(C_6_F_5_)_2_)_2_C_6_F_4_/(Et_3_SiH) in which hydride was immediately removed from the silane to form a hydridoborate/silylium complex. Acetophenone was introduced to one of the catalytic pairs along with triethylsilane, producing the exhaustively reduced ethylbenzene/ hexaethyldisiloxane in a 1:1 mixture to unreacted carbonyl. In contrast, tris(pentafluorophenyl)-borane catalyst had generated the silyl ether preliminary reduction product “exclusively”. The alternative reductive pathway was proposed to proceed through a unique mechanism in which the carbonyl first binds to the silylium species, creating an activated substrate reduced by hydride transfer from triethyl silane to produce silyl ether and regenerate the silylium catalyst. The newly formed silyl ether oxygen attacks the developing silylium, forming a disilyl ether oxonium in which the disilyl ether, Et_3_SiOSiEt_3_, readily dissociates to form a transient carbenium intermediate reduced *via* silyl hydride abstraction to produce the fully deoxygenated product.

### 13.2. Conclusive Evidence for an S_N_2-Si Mechanism

**Figure 36:**
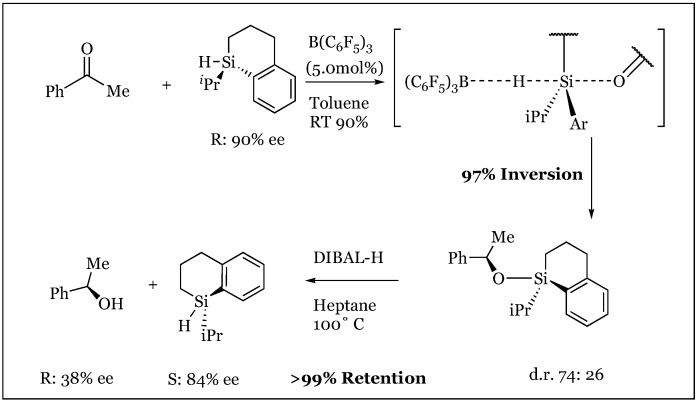
Walden inversion observed in the conversion of the *R* to *S* enantiomer of the silane stereocenter in the hydrosilylation of acetophenone.

**Figure 37:**
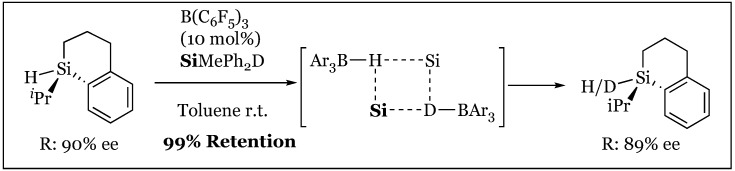
Isotopic H/D scrambling in the presence of tris(pentafluorophenyl)borane with silane retention via four-membered transition state.

The fate of the silane-borane complex was elucidated through an elegant Walden inversion, culminating in an S_N_2 displacement of the borane by the attacking nucleophilic substrate [77]. Investigation of a potential silane partner noted complete absence of reactivity in sterically encumbered silanes with a *tert*-butyl substituent. Thus, further experimentation was conducted with an isopropyl substituted, 90% enantioenriched silane in the presence of enriched prochiral acetophenone and the tris(pentafluorophenyl)borane catalyst. A diastereomeric mixture of the silyl ether adduct was obtained (d.r. 74:26) under the reaction conditions. This mixture was reductively cleaved with DIBAL-H maintaining the configuration at silicon-observing 97% inversion to S at silicon and a significant 38% enantiomeric excess of the generated alcohol (Figure 36). It is thus speculated that interaction of acetophenone with chiral silicon favors hydride transfer to the *si* face. An S_N_1 mechanism proceeding through an achiral silylium ion was negated, as a racemic mixture of products would result. In addition, combination of enantioenriched *R* silane with deuterated achiral silane treated with B(C_6_F_5_)_3_ revealed complete isotopic dispersion as well as the telling conservation of stereochemistry about the chiral silane (Figure 37). Thus, a nucleophilic attack anti to a Si-H-B bridging arrangement was proposed, potentially proceeding through a four-membered transition state that would permit hydrogen exchange. 

### 13.3. Direct Observation of a Borane-Silane Complex

**Figure 38:**
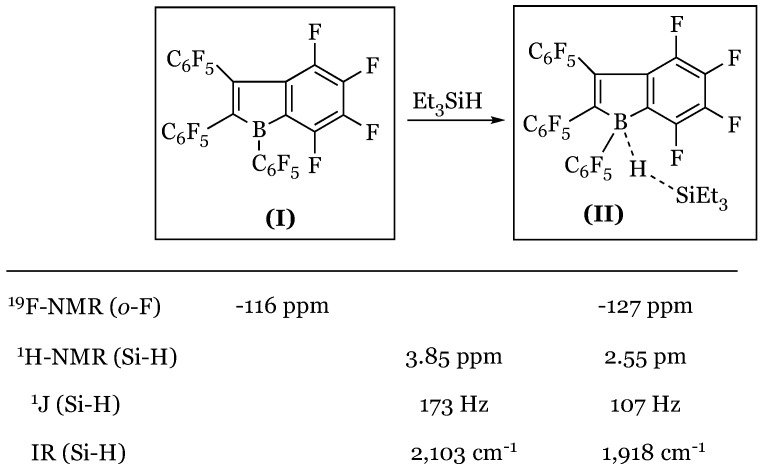
Spectral evidence for borane-silane complex formation.

Conclusive evidence for borane’s activation of the Si-H bond was obtained by direct characterization of the respective complex collectively by Piers and Tuononen [77]. A modified borane catalyst with increased Lewis acidity was synthesized, (1,2,3-tris(pentafluorophenyl)-4,5,6,7-tetrafluoro-1-boraindene) (Figure 38, (**I**)) and treated with triethylsilane.

Unique to this Lewis acid is a characteristic red hue at 465 nm which shifts to yellow as it undergoes complex formation. Thus, a visible indication of successful silane-borane interaction (Figure 38, (II)) was observed upon lowering the temperature to 195 K. ^19^F-NMR spectroscopic analysis also demonstrated a significant upfield shift of the fluorine *ortho* to the boron center proportional to decreasing temperature as well as to varying silane concentration. Weakening of the Si-H bond was evident in diminishing values of the J_Si-H_ coupling constant from 177 to 107 Hz, as well as in an infrared shift in the Si-H bond from 2103 cm^−1^ to 1918 cm^−1^ upon complex formation (Figure 38). Proposal of an Si-H-B bridge was based on observed distortions from free Si-H (≈1.48 Å) and B-H (≈1.14 Å) to 1.51 Å and 1.46 Å, by X-ray crystallography, respectively, indicative of hydride abstraction as the Si-H bond lengthens.

## 14. Quantum Chemical Calculations

### 14.1. Stable Complex Between Trimethyl silane and B(C_6_F_5_)_3_

**Figure 39:**
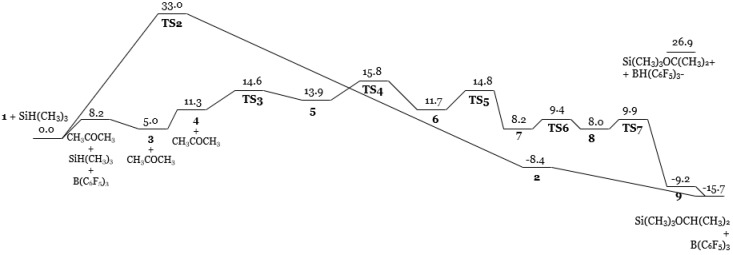
Quantum chemical calculations of the reaction coordinate diagram for the reduction reaction of acetone.

**Figure 40:**
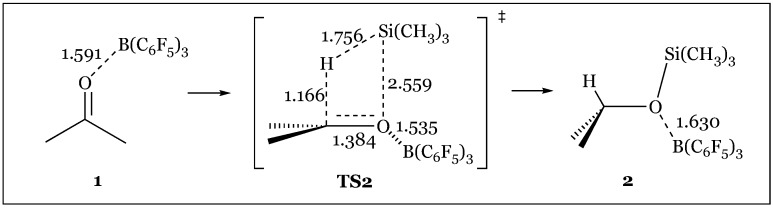
Intermediates and transition states of the carbonyl-activation reduction mechanism.

**Figure 41:**
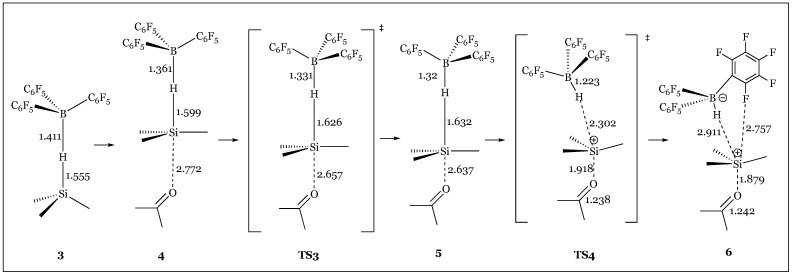
Intermediates and transition states of the silane-activation reduction mechanism.

Quantum chemical calculations comparing the proposed catalytic activities of the borane catalyst- the canonical carbonyl activation pathway versus the Frustrated Lewis Pair strained priming of the Si-H bond for heterolytic cleavage- supported that the latter is more energetically favorable (Figure 39) [78]. Carbonyl activation initially seems plausible because in solution, a complex between B(C_6_F_5_)_3_ and acetone is 8.2 kcal/mol lower in free energy than the liberated species. This proposed pathway is concerted as only one key four-membered transition state is observed upon attack of the silane to the carbonyl moiety. This affords an oxonium species bound to both the silyl and borane catalytic components, albeit the energetics of the transition state are disconcertingly high at 33.0 kcal/mol (Figure 40). Facile dissociation of the borane catalyst alleviates the steric strain accompanying the aryl substituents and consummates the process yielding the final silyl ether product. In contrast, the silyl activation pathway proceeds through five transition states, beginning with the silane-borane adduct via a hydride bridge requiring a mere 3.2 kcal/mol complexation energy (Figure 41). As the carbonyl attacks the backside of the quasi linear Si-H-B array, the original Si-H and B-H bond distances of 1.555 and 1.411 Å respectively are distorted as hydride abstraction by the Lewis acid is enabled by a “pushing” of the Si-H bond by the carbonyl moiety (Figure 41). Free energies of 14.6 (TS3) and 15.8 (TS4) kcal/mol of the transition states involving the initial stages of carbonyl attack are significantly lower than the transition state elaborated in the carbonyl activation mechanism. Dissociation into the hydridoborate and silyloxonium species is observed, which remain as a close contact ion pair through the final three transition states- “rotation of the acetone plane” (TS5), “transformation into another arrangement of the ion-pair complex” (TS6), with the awaited hydride transfer from the borane anion to the carbonyl carbon concluding the reduction pathway (TS7). Further calculations comparing B(C_6_F_5_)_3_ to frequently used Lewis acid catalysts BF_3_ or BCl_3_ which operate via a carbonyl activation pathway has revealed that the unique catalytic pathway of tris(pentafluorophenyl)borane is characterized by a stronger Lewis Acidity towards the silane which is “transferred to the reactive orbital of silane” strengthening the carbonyl’s nucleophilic attack. Further, interactions between the ortho fluorine atoms of the borane catalyst with the silane center stabilize the unfavorable deformations required to form the frustrated pair Si-H-B complex. 

### 14.2. Imines via Silyliminium Intermediates

**Figure 42:**
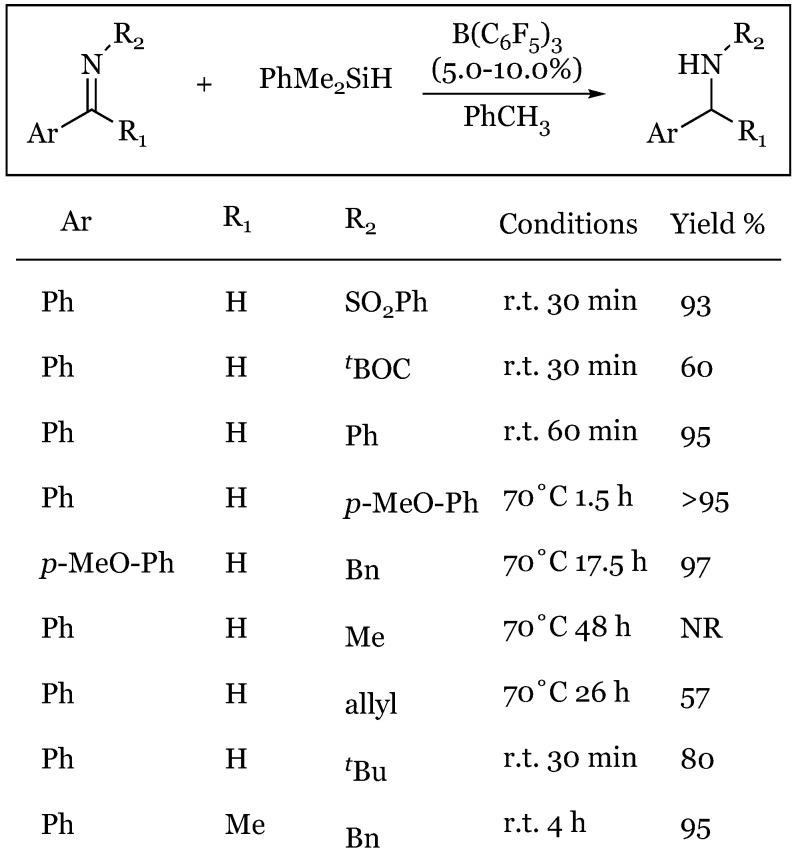
Reaction rate and yield in the reduction of imines based on substituents.

Study of imine reduction has revealed an analogous silane-activation mechanism proceeding through a silyliminium/hydridoborate ion pair [79]. The function of the tris(pentafluorophenyl)-borane catalyst as an activator of the Si-H bond was deduced based on the effect of the basicity of nitrogen on reaction rate (Figure 42). It was found that through variance of the *N*-substituent, the rate of reaction was easily manipulated. Powerful electron withdrawing groups such as SO_2_Ph “which prevented imine complexation to B(C_6_F_5_)_3_ as observed in ^19^F-NMR” and *tert*-butyloxocarbonyl were reduced most rapidly. Reaction rates increase with substituent size in the series *tert*-butyl >> benzyl >> allyl >> methyl presumably due to the increasing impediment to interaction with the boron Lewis acid. No reduction occurs in the presence of strong complex formation, as evident in the methyl substituent, as free borane is required for catalytic silane activation. Spectroscopic data obtained from ^19^F-, ^11^B-, ^29^Si-, and ^1^H-NMR support the formation of a silyliminium-hydridoborate ion pair upon hydrogen abstraction from the silane, concurrent with imine’s nucleophilic attack of silane. The hydridoborate anion is observed in ^19^F-NMR even as the reaction is consummated in the formation of amine product, implying imine activation may be effected by the PhMe_2_Si cation rather than by B(C_6_F_5_)_3_.

### 14.3. Refined Imine Hydrosilylation Mechanism Utilizing a Chiral Borane

**Figure 43:**
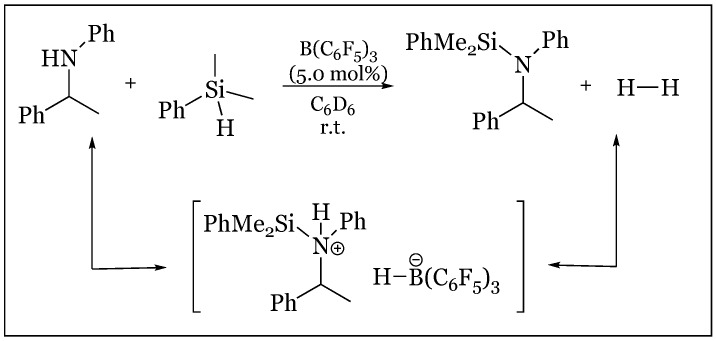
Nitrogen-silyl coupling mediated by tris(pentafluorophenyl)borane.

**Figure 44:**
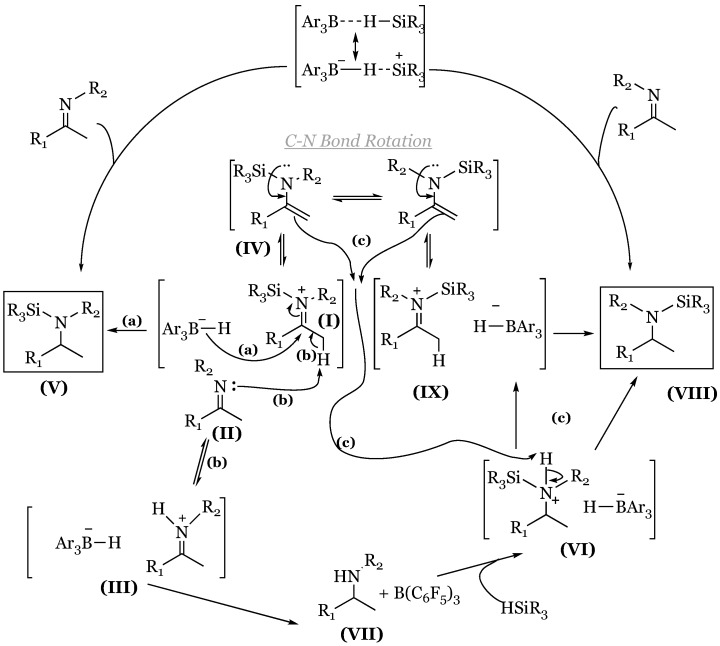
Refined mechanism of reducing *N*-substituted imines bearing an α-hydrogen atom to *N*-silylated amines: (**a**) hydridoborate reduction of *N*-silylated iminium ion; (**b**) loss of proton from *N*-silylated iminium ion to give iminium ion (III) and enamine (IV); (**c**) deprotonation of *N*-silylated ammonium ion to give silyl iminium ion (IX) and *N*-silylated amine (VIII).

The mechanism of imine hydrosilylation was conclusively studied using uncharacteristically small loadings of B(C_6_F_5_)_3_ (approximately 0.1 mol%), lowering the reaction rate to enable the observation of unexpected key intermediates [80]. An allusion to a unique reaction pathway was initially detected within the imine reduction of 1,1-methylphenyl *N*-benzyl imine with a chiral borane utilized as a stereochemical probe to confirm the presumed method previously elucidated in carbonyl reduction. However, accompanying the production of *N*-silylated amine, unexpected intermediates of *N*-silylated enamine as well as free amine were observed in equimolar ratios, based on characteristic ^13^C NMR peaks. Curiously, these intermediates were more pronounced at high concentrations of THF, and hindered the overall reaction rate. The free amine was explicated with an equilibrium between the imine and protonated THF, producing an iminium ion that was subsequently reduced to the free amine by the hydridoborate anion. Conversely, enamine formation occurred in the deprotonation of the silyliminium ion via THF or unreacted imine acting as a BrØnsted base. The latter process would explain equimolar intermediate formation, as deprotonation induced by the imine produces the iminium predecessor to the free amine intermediate. The same intermediates at equimolar ratios were observed with catalysis of B(C_6_F_5_)_3_ at markedly low loadings (approximately 0.1%). Conversion of the free amine to the *N*-silylated product was achieved via Si-N coupling mediated by the boron catalyst, proceeding through a unique ammonium-hydridoborate ion pair (Figure 43). The remarkable stability of this ion complex is hypothesized to proceed through an alternative route in which ammonium protonates the *N*-silylated enamine intermediate, to produce the *N*-silylated amine and silyliminium ion, respectively. Intermediate formation was further supported in a labeling experiment in which the methyl group of the imine starting material was fully saturated with deuterium which was then transferred to the nitrogen of the free amine concurrent with enamine formation. Final reduction by the hydridoborate ion via silane activation was confirmed with the inclusion of deuterium, which resulted in deuterium’s final placement at the tertiary carbon of the product. The revised mechanism including the deprotonation/protonation pathways is depicted below (Figure 44). The *N*-silylated iminium ion (**I**) may lose an alpha proton to free imine (**II**), generating an iminium ion (**III**) and the enamine intermediate (**IV**) denoted by pathway (b), or may undergo hydridoborate reduction to form *N*-silylated amine (**V**) denoted by pathway (a). Potential enamine-mediated proton removal from the *N*-silylated ammonium ion (**VI**) forms the silyl iminium ion (**IX**) and the *N*-silylated amine (**VIII**).

### 14.4. Proposed Mechanism for the Exhaustive Reduction of Alcohols and Ethers

**Figure 45:**
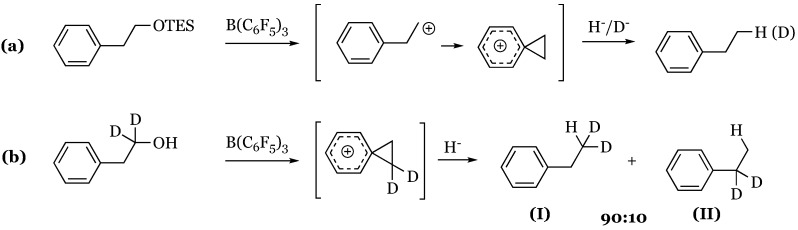
Postulated steps of reduction of: (**a**) phenylethanol-triethylsilyl ether *via* phenonium cation; (**b**) 1,1-dideuteriophenethyl alcohol via dideuteriophenonium cation (invalidated due to 90:10 product distribution).

**Figure 46:**
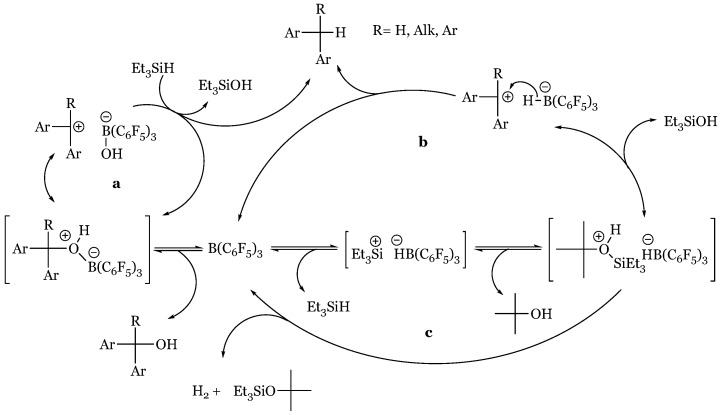
Suggested S_N_1 pathways for alcohols with: (**a**) and (**b**) carbocation-supporting aryl substituents; (**c**) dehydro-condensation to silyl ether for less-stabilized alcohols.

**Figure 47:**
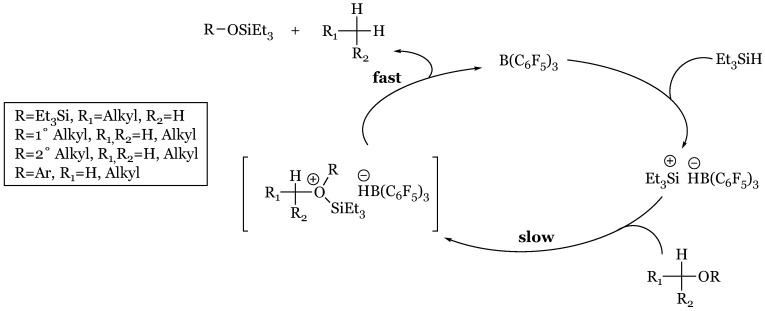
Exhaustive reduction of silyl ether generated by initial dehydro-condensation to hydrocarbon.

Reduction of primary alcohols in high yield has been observed with excess Et_3_SiH and catalytic loadings of tris(pentafluorophenyl)borane catalyst [27]. Successful transformation has also been achieved with HSiPh_3_, HSiMePh_2_, and HSiMeEt_2_. In contrast, secondary and tertiary alcohols yield silyl ethers with the exception of di- and triphenylmethanol, which are effectively reduced to hydrocarbons. While the lack of reduction of secondary and tertiary alcohols suggests an S_N_2 mechanism, conflicting results were observed in the treatment of secondary *S*-methylphenyl silyl ether with DSiEtMe_2_ and B(C_6_F_5_)_3._ For the latter substrate, the expected inversion of configuration (41% ee) was obtained only in pentane. Enhancing the dielectric constant and the potential to support a carbocationic intermediate with the use of dichloromethane produced a racemic mixture with negligible enantiomeric excess of 2% retention. Further analysis of the reduction of primary alcohols utilizing the model system of phenylethanol-triethylsilyl ether, 1:1 HSiEt_3_ to DSiEt_3,_ and 10 mol% B(C_6_F_5_)_3_ exhibited no primary kinetic isotope effect that would be expected in an S_N_2 mechanism in which reductive transfer of hydride/deuteride would occur in the rate determining step suggesting an S_N_1 pathway stabilized by the phenonium cation intermediate (Figure 45a). However, unexpected product distributions (90 I:10 II rather than equimolar) of the analogous deuterated alcohol- 1,1-dideuteriophenethyl alcohol- negated an S_N_1 pathway in this case (Figure 45b). Further kinetic study of 1,1-dideuteriophenethyl alcohol reduction determined facile formation of the silyl ether was followed by a laborious slow reduction to form the hydrocarbon. Based on this mechanistic disagreement, it was inferred that the reduction of alcohols possessing carbenium stabilizing groups likely proceeds through two possible S_N_1 routes (Figure 46) while less labile alcohols proceed through the aforementioned two step pathway (Figure 46 and Figure 47).

Pathway A begins with the dative oxonium complex between the alcohol and borane Lewis acid (Figure 46), which dissociates into the respective carbocation. This carbocation is reduced by the silane to form the hydrocarbon product, silyl alcohol, and regenerated catalyst. Pathway B is similar to carbonyl and imine reduction pathways, initially forming the silyl-borane frustrated pair from which the silyl group is transferred to the alcohol to form a silyloxonium-hydriodoborate complex. Silyl alcohol disengagement generates the transient carbocation, energetically reclaimed by hydride transfer via hydridoborate to the stable hydrocarbon product. Alcohols devoid of electron donating substituents also interact with the silyl-borane pair to form an oxonium ion; however, at this point dehydro-condensation forms the silyl ether, consuming one equivalent of silane (Figure 46). Exhaustive reduction may occur in the presence of excess silane as the silyl ether attacks the silyl substituent from the silyl-borane adduct to form a second oxonium ion, reduced by hydridoborate (Figure 47). Silyl transfer from the silyl-borane adduct to the silyl ether is rate determining due to steric concerns in the coordination, rectifying the near-exclusive preference for primary alcohol reduction.

## 15. Conclusions

The tris(pentafluorophenyl)borane-silane Frustrated Lewis pair has been demonstrated in its broad range of reductive applications across a multitude of functional groups including aldehydes and ketones [54], imines [61], amides [33], nitriles [49], olefins [5], ethers [27], and carboxylic acid derivatives [29] to be an invaluable alternative to harsh metallic conditions or its boron trihalide alternatives. Its catalytic proficiency under mild aqueous conditions has rendered its utilization irresistible and led to its burgeoning demand as a potent catalyst uniting organic and inorganic spheres. The strained nature of the silyl-borane hydride bridging system has enabled its wide breadth of exploitation as strategic manipulation of silyl or reagent substituents permits stereoselectivity. This is exemplified in asymmetric induction reliant on sigma-pi chelation of alkynyl systems [55], manipulation of stereochemistry dependent on steric hindrance about the silane [56], as well as *anti*-addition across olefins [5]. Regiochemical control has been exhibited in preferential placement of the silylium species at the terminal carbon of olefins [5], reduction of the terminal carbon concurrent with hydrosilylation of the internal carbon in the deoxygenation of 1,2 diols [40], as well as the preferential addition to the beta carbon of internal ynamides [50]. Further, tris(pentafluorophenyl)borane silane catalysts may bear promising applications in synthetic pathways requiring functional group selectivity, perhaps eliminating the need for cumbersome protection-deprotection steps, as exhibited in the exclusive reduction of the olefin within conjugated esters and amides [47] and the reduction of nitriles in the presence of nitro, halogen, and olefin groups [49]. The extent of the reaction towards full deoxygenation may be halted at the preliminary silyl ether with intentional addition of one molar equivalent of silane [22,30] due to the stoichiometric nature of the reduction. Incumbent to the findings presented in this review are continued pursuit of optimal conditions for reductions yet to proceed in high yields such as aliphatic amides [52]. We propose continued development of the tris(pentafluorophenyl)borane catalyst will rely on the selection of appropriate silane in regards to its stereoelectronic characteristics to achieve efficient reduction with stereo-, regio-, and functional selectivity.
